# Supplementary material for: Collaborating to Improve Neonatal Care: ParentAl Participation on the NEonatal Ward—Study Protocol of the neoPARTNER Study
Source: Children (Basel). 2023 Aug 30;10(9):1482. doi: 10.3390/children10091482 (PMC10527908; doi:10.3390/children10091482)
Supplement: Supplementary file 1 [file children-10-01482-s001.zip › children-2496454-supplementary.pdf]

Supplementary Material—Table S1

Table S1. List of additional members of the neoPARTNER study and project group.

Author's Name	Affiliation/Participating Centre	City	Role
Cécile R.L. Boot	Department of Public and Occupational Health, Amsterdam UMC, VU University<br><br>Amsterdam Public Health research institute, Societal Participation and Health	Amsterdam, The Netherlands	Member of neoPARTNER project group[1]
George Damhuis	Division of Neonatology, Department of Paediatrics, Erasmus MC Sophia Children's Hospital, University Medical Centre Rotterdam	Rotterdam, The Netherlands	Member of neoPARTNER project group[1]
Nanon H.M. Labrie	Department of Language, Literature & Communication, Amsterdam UMC, VU University	Amsterdam, The Netherlands	Member of neoPARTNER project group[1]
Enrico Lopriore	Division of Neonatology, Department of Paediatrics, Leiden University Medical Centre	Leiden, The Netherlands	Member of neoPARTNER project group[1]
Sylvia A. Obermann-Borst	Care4Neo, Neonatal Patient and Parent Advocacy Organization	Rotterdam, The Netherlands	Member of neoPARTNER project group[1]
Marijn J. Vermeulen	Care4Neo, Neonatal Patient and Parent Advocacy Organization<br><br>Division of Neonatology, Department of Paediatrics, Erasmus MC Sophia Children's Hospital, University Medical Centre Rotterdam	Rotterdam, The Netherlands	Member of neoPARTNER project group1
Ron H.T. van Beek	Amphia Ziekenhuis	Breda, The Netherlands	Paediatrician-neonatologist, local PI

Bas Harzing	Amphia Ziekenhuis	Breda, The Netherlands	Research assistant
Hannie A.M.M. van Achterberg	Amphia Ziekenhuis	Breda, The Netherlands	Research assistant
Lotte H. Hendrikx	BovenIJ Ziekenhuis	Amsterdam, The Netherlands	Paediatrician, local PI
Ariena J. Rasker[2]	BovenIJ Ziekenhuis	Amsterdam, The Netherlands	Research assistant
Okke Hoonhout[2]	BovenIJ	Amsterdam, The Netherlands	Research assistant
Claire A.M. Lutterman	Flevoziekenhuis	Almere, The Netherlands	Paediatrician-neonatologist, local PI
Jessica E. van Veen	Flevoziekenhuis	Almere, The Netherlands	Research assistant
Romee M. Willemsen	Flevoziekenhuis	Almere, The Netherlands	Research assistant
Angelique K.E. Hoffmann-Haringsma	Franciscus Gasthuis & Vlietland	Rotterdam, The Netherlands	Paediatrician-neonatologist, local PI
Mariëlle Maissan	Franciscus Gasthuis & Vlietland	Rotterdam, The Netherlands	Physician-assistant
Anne M. de Grauw	Juliana Kinderziekenhuis	Den Haag, The Netherlands	Paediatrician-neonatologist, local PI
Leo Wewerinke	Juliana Kinderziekenhuis	Den Haag, The Netherlands	Paediatrician-neonatologist, local investigator
Alma Qureshi	Juliana Kinderziekenhuis	Den Haag, The Netherlands	Research assistant
Ageeth G. Kaspers	Medisch Spectrum Twente	Enschede, The Netherlands	Paediatrician-neonatologist, local PI
Deborah Zagers	Medisch Spectrum Twente	Enschede, The Netherlands	Research assistant
Femke de Groof	Noordwest Ziekenhuisgroep Alkmaar	Alkmaar, The Netherlands	Paediatrician-neonatologist, local PI
Priscella Eppenga-Franklin	Noordwest Ziekenhuisgroep Alkmaar	Alkmaar, The Netherlands	Research assistant
Monique J.M. van Brakel	Tergooi MC	Hilversum, The Netherlands	Paediatrician, local PI
Celine Seegers	Tergooi MC	Hilversum, The Netherlands	Research assistant
Maarten Rijpert	Zaans Medisch Centrum	Zaandam, The Netherlands	Paediatrician-neonatologist, local PI
Esther Scheijbeler[2]	Zaans Medisch Centrum	Zaandam, The Netherlands	Research assistant

Fenna Visser	Ziekenhuis Amstelland	Amstelveen, The Netherlands	Paediatrician, local PI
Koen Ruiter[2]	Ziekenhuis Amstelland	Amstelveen, The Netherlands	Research assistant
Esther J. Kret[2]	Ziekenhuis Amstelland	Amstelveen, The Netherlands	Research assistant

PI = principal investigator

[1] Members of the neoPARTNER project group greatly contributed to the design of the study and the preparation of the original study protocol.

[2] Primarily employed at OLVG, detached to participating centre during course of the study.
